# Fossil clam shells reveal unintended carbon cycling consequences of Colorado River management

**DOI:** 10.1098/rsos.160170

**Published:** 2016-09-28

**Authors:** Jansen A. Smith, Daniel A. Auerbach, Karl W. Flessa, Alexander S. Flecker, Gregory P. Dietl

**Affiliations:** 1 Department of Earth and Atmospheric Sciences, Cornell University, Ithaca, NY 14853, USA; 2 Department of Ecology and Evolutionary Biology, Cornell University, Ithaca, NY 14853, USA; 3 Department of Geosciences, University of Arizona, Tucson, AZ 85721, USA; 4 Paleontological Research Institution, Ithaca, NY 14850, USA

**Keywords:** carbon emission, carbon sequestration, estuary, geohistorical records, mollusc, water diversion

## Abstract

Water management that alters riverine ecosystem processes has strongly influenced deltas and the people who depend on them, but a full accounting of the trade-offs is still emerging. Using palaeoecological data, we document a surprising biogeochemical consequence of water management in the Colorado River basin. Complete allocation and consumptive use of the river's flow has altered the downstream estuarine ecosystem, including the abundance and composition of the mollusc community, an important component in estuarine carbon cycling. In particular, population declines in the endemic Colorado delta clam, *Mulinia coloradoensis*, from 50--125 individuals m^−2^ in the pre-dam era to three individuals m^−2^ today, have likely resulted in a reduction, on the order of 5900–15 000 t C yr^−1^ (4.1–10.6 mol C m^−2^ yr^−1^), in the net carbon emissions associated with molluscs. Although this reduction is large within the estuarine system, it is small in comparison with annual global carbon emissions. Nonetheless, this finding highlights the need for further research into the effects of dams, diversions and reservoirs on the biogeochemistry of deltas and estuaries worldwide, underscoring a present need for integrated water and carbon planning.

## 1. Introduction

Rivers worldwide have been profoundly modified to maximize the production of a subset of the services they provide, such as hydroelectric power and reliable water for irrigation and municipal use [1,2]. The upstream infrastructure and water diversions required to provide these services can, however, have a pronounced effect on downstream deltas and estuarine ecosystems. With human demands for freshwater remaining high or increasing, deltas and estuaries are poised to experience heightened stress from ocean acidification and sea-level rise as the climate changes. These compounded stressors may have wide reaching impacts, including perturbing carbon cycling in river systems [3,4]. Yet, our current understanding of the influence of large-scale water management on carbon sequestration and emission hinders the comprehensive evaluation of infrastructure or other water resource planning [5–7].

**Figure 1.**
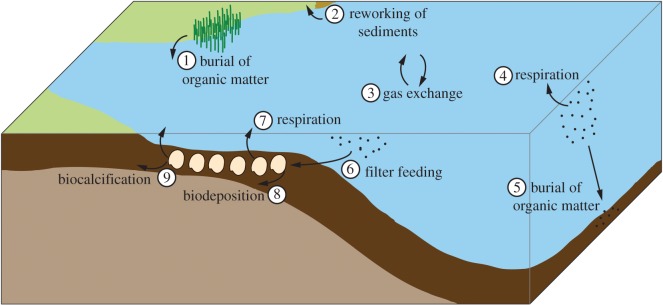
A subset of the processes involved in estuarine carbon cycling. (1) Sequestration of carbon via vegetation (e.g. salt marshes, mangroves) growth, death and burial; (2) emission of carbon due to reworking of carbon-rich sediments; (3) constant gas exchange between ocean and atmosphere; (4) emission of carbon via respiration by microbes and zooplankton; (5) sequestration of carbon via burial of dead plankton; (6) filter feeding by bivalves; (7) carbon emission via bivalve respiration; (8) carbon sequestration via biodeposition and (9) carbon sequestration and emission via biocalcification.

The need for research examining the carbon consequences of water management associated with the alterations of biophysical processes in rivers is particularly acute in deltas and their estuaries [3]. Carbon emissions from estuaries, which can range from 17 to 46 mol C m^−2^ yr^−1^ [8], are significant in regional carbon budgets [9,10] and cumulatively can amount to global emissions of 3.4–4.5 × 10^8^ t C yr^−1^ [8,11]. Whereas overall emissions from estuaries are becoming well quantified [12], the individual components of the multifaceted estuarine carbon cycle (figure 1) merit further study, particularly molluscs.

Molluscs are a vital element of the tidal and subtidal benthic fauna of estuaries, collectively emitting 2–20 mol C yr^−1^ [13–15] and playing a large role in the pelagic–benthic cycling of nutrients (e.g. carbon, nitrogen, phosphorus [16–19]). In particular, bivalve molluscs (commonly, ‘clams’) can be found locally in densities of more than 1000 individuals m^−2^ [20,21] and can contribute significantly to estuarine carbon emissions via respiration and biogenic calcification (i.e. shell formation). Although carbon sequestration exceeds carbon emission during shell formation, the amount of carbon dioxide released via respiration typically exceeds the amount of carbon that is sequestered, resulting in net carbon emissions [13–15,22]. It is well documented that water management can affect molluscan populations [4,23,24]; however, to the best of our knowledge, the carbon implications of these effects have never been assessed.

**Figure 2.**
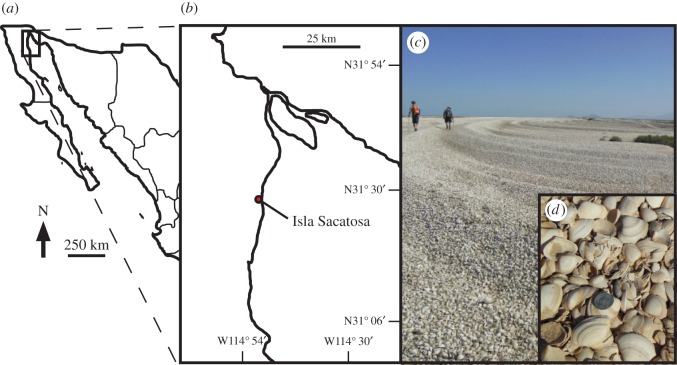
Cheniers in the Colorado River delta. (*a*) Location of delta in Mexico. (*b*) Colorado River delta locality from Kowalewski *et al*. [23]. (*c*) A chenier in the Colorado River delta at a locality south of Isla Sacatosa. (*d*) Close-up of *Mulinia coloradoensis*; coin, 0.24 cm in diameter.

The palaeoecological record on the Colorado River delta (CRD; figure 2) offers a unique means to conduct such an analysis. Prior to damming and diversions in the 1930s, natural annual flows ranged between 16 × 10^9^ and 18 × 10^9^ m^3^ at Lee's Ferry in northern Arizona [26], but the river now fails to reach the sea in most years (with the exception of the recent environmental pulse flow under the groundbreaking binational Minute 319 agreement [27]). The lack of Colorado River water has led to substantial declines in clam [23], shrimp [28] and fish [29] populations, in addition to reduction in estuarine, riparian and wetland vegetation [30]. Salt marshes, wetlands and riparian forests now cover a small fraction of their former area [31,32], and the once brackish estuary is now saltier than the sea [33,34].

Very few environmental data were collected before the main period of dam construction (1900–1965) on the Colorado River. However, palaeoecological data from cheniers—beach ridges composed almost exclusively of molluscan remains [35,36]—permit estimates of the size and density of the pre-dam (prior to 1930) molluscan community, particularly the endemic Colorado delta clam, *Mulinia coloradoensis* [23] (figure 2). Cheniers of three ages are present, two that formed between 90–1500 and 2000–5000 years ago, and a third that has been actively forming over the past 90 years [25]. Dated shells from these cheniers indicate that *M. coloradoensis* accounted for as much as 95% of the individuals during the pre-dam era and occurred at densities that were likely well more than 50 individuals m^−2^ [23]. In contrast, current molluscan densities of 3–17 individuals m^−2^ [37] indicate the scope of the ecological change associated with river management [23,24,38]. Accordingly, we combined evidence of pre-dam conditions from cheniers with data from contemporary mollusc community surveys to investigate how reduced clam density following upstream river diversion altered carbon sequestration and emissions (in terms of CO_2_) in the estuary of the CRD.

## 2. Methods

### 2.1. Clam density

We established the estimates for the density of clams living on the CRD tidal flats during the pre-dam era and the present day based on the work of Kowalewski *et al.* [23] and Cintra-Buenrostro *et al.* [38]. Kowalewski *et al*. [23] estimated pre-dam clam density for the 90–1500 year old chenier by combining bulk samples from the molluscan assemblage, estimates of the total area of shell deposits and an average chenier thickness (table 1). They estimated an average density of 87 500 clams m^−3^, which translates to approximately 2.1 × 10^12^ total clams in the chenier. Amino acid racemization confirmed that 98% (*n* = 125) of the shells in the chenier dated from the period C.E. 950–1950. Based on estimates of the pre-dam tidal flat area (table 1) and an average ontogenetic age of three years, Kowalewski *et al*. [23] then calculated an average clam density of approximately 50 individuals m^−2^ during this period. This value likely represents a conservative estimate of clam density given that various taphonomic processes (e.g. shell dissolution, abrasion, fragmentation, etc.) remove shell material after death [25,44]. Based on the age distribution of dated shells and accounting for shell removal, Kowalewski *et al.* [23] proposed that the total number of clams alive during C.E. 950–1950 may have exceeded 5 × 10^12^, or a constant standing density of 125 individuals m^−2^ across the entire CRD tidal flat. This estimate remains highly conservative, however, because, for practical reasons, Kowalewski *et al.* [23] did not consider individuals smaller than the 12.5 mm mesh size that they used to sieve samples. Indeed, surveys of the living community suggest that large individuals (more than 12.5 mm) may compose the majority of biomass yet represent only 20% of individuals in the community [23]. Thus, we calculated changes in carbon based on pre-dam densities of 50 and 125 individuals m^−2^ in order to estimate a conservative range of values.

**Table 1.** Parameter estimates and assumptions for carbon calculations. AFDM, ash-free dry mass.

parameter	estimate	source
chenier area	5.96 × 10^6^ m^2^	Kowalewski *et al*. [23]
chenier thickness	4 m	Kowalewski *et al*. [23]
chenier volume	2.4 × 10^7^ m^2^	Kowalewski *et al*. [23]
clams by volume in cheniers	87 500 m^−3^	Kowalewski *et al*. [23]
tidal flat area	1.2 × 10^8^ m^2^	Kowalewski *et al*. [23]
pre-dam clam density	50–125 ind m^−2^	Kowalewski *et al*. [23]
modern clam density	3–17 ind m^−2^	Kowalewski *et al*. [23], Avila-Serrano *et al*. [37]
clam dry mass	11.13 ± 8.3 g ind^−1^	Measured (electronic supplementary material, table)
pre-dam salinity (north)	22–32 psu	Cintra-Buenrostro *et al*. [39]
pre-dam salinity (south)	30–38 psu	Cintra-Buenrostro *et al*. [39]
modern salinity	35–42 psu	Dettman *et al*. [40]
dry tissue weight	1.36 g ind^−1^	Velasco & Navarro [41]
dry tissue (DT) to AFDM	1 g DT = 0.81 g AFDM	Rumohr *et al*. [42]
AFDM gram to kilocalorie	1 g AFDM = 5.492 kcal	Cummins & Wuycheck [43]
kilocalorie to grams carbon	11.4 kcal = 1 g C	Chauvaud *et al*. [13]

Surveys of the contemporary tidal flat molluscan community along seven transects throughout the CRD suggest an average density of approximately 17 individuals m^−2^ [23,37]. This estimate includes molluscs of all sizes; however, when only considering clams greater than 12.5 mm, density drops to three individuals m^−2^ [23]. We based present-day values on this latter estimate for consistency with the pre-dam data.

### 2.2. Carbon storage and emission

Using these pre-dam and present-day estimates of clam density, we then calculated annual carbon sequestration and production from biogenic calcification and carbon emission due to respiration. Annual carbon sequestration was calculated based on standing clam density and one-third average shell dry mass (assuming constant growth over a clam's three-year lifespan for ease of calculation; table 1), with mass corrected to account for the approximately 5% of compounds other than calcium carbonate in clam shells [45].

$$\text{calcium carbonate}\;\left(\frac{\text{g}}{{\text{m}}^{2}}\right)=\text{clam density}\;\left(\frac{\text{clams}}{{\text{m}}^{2}}\right)\times \left[\frac{\left(\text{shell dry mass}\;(\text{g per clam})\times 0.95\right)}{3}\right]$$

We determined average shell dry mass (11.13 ± 8.3 g ind^−1^) by taking the mass of 100 specimens of *M. coloradoensis* (electronic supplementary material, table S1), which composed as much as 95% of the molluscan community in the pre-dam era [24,25]. The *M. coloradoensis* specimens were randomly selected from a bulk sample [46] that was sieved with a 12.5 mm screen to ensure comparability with the data reported by Kowalewski *et al.* [23]. We estimated grams of carbon per square metre by adjusting for the atomic weight of calcium carbonate:

$$\text{carbon}\;\left(\frac{\text{g}}{{\text{m}}^{2}}\right)=0.12\times \text{calcium carbonate}\;\left(\frac{\text{g}}{{\text{m}}^{2}}\right)$$

We then multiplied g C m^−2^ by the total area of the tidal flat to provide values of annual average sequestration (table 1).

We calculated the production of carbon due to biogenic calcification according to the ratio of released (CO_2_) to precipitated (CaCO_3_) carbon (*ψ* [47]). Carbon emission to the atmosphere during biogenic calcification is buffered by water chemistry, with the exchange rate influenced by temperature and salinity. Higher temperatures and greater salinities reduce *ψ*, decreasing the emission of CO_2_ to the atmosphere relative to the precipitation of carbonate (i.e. fresh, cold water leads to the least precipitation). Seawater temperature data are available for the present day and vary seasonally from 5 to 40°C [48,49], but data from the pre-dam era are limited to a handful of data points at 18–20°C, collected by the US Fish Commission steamer *Albatross* in March 1889 [50]. Owing to this limitation on temperature data, we used the univariate *ψ* equation for salinity, which does not include temperature (i.e. assumes constant temperature)

$$\psi =0.949-(7.9\times 10^{-3}\times S),$$

where *ψ* is the ratio of released : precipitated carbon, and *S* is salinity in practical salinity units (psu), and *p*CO_2_ = 350 µatm^1^ [13,15,22,47].

Salinity in the CRD has increased following the construction of dams throughout the basin [24,33]. Today, salinities range from 35 to 42 psu [40], though periodic flow releases (at significantly lower volumes than occurred historically) have occasionally reduced salinities to 29–36 psu [40,51]. We therefore assumed a value of 38.5 psu for the present. Isotopic data from the shells of *M. coloradoensis* suggest individuals near the mouth of the river grew under salinities of 22–33 psu, whereas individuals further south experienced salinities of 30–38 psu [39]. We used the mean value from the isotopic data, 30 psu, which is in the range reported during more recent flows, to calculate changes in carbon emissions.

Finally, carbon emitted due to respiration was calculated using the relationship established by Schwinghamer *et al.* [52]:

$$\log_{10}R=0.367+0.993\log_{10}P,$$

where *R* and *P* are respiration and biomass production (kcal m^−2^ yr^−1^), respectively. We applied the conversion factor of 5.492 kcal = 1 g ash-free dry mass (AFDM) to estimate *P* [43] and estimated carbon emission from *R* as 11.4 kcal = 1 g C [13]. Live *M. coloradoensis* were not available to determine AFDM; however, dry tissue weights of 1.36 g individual^–1^ have been reported in the literature for the closely related and morphologically similar *Mulinia edulis* [41] and can be converted to AFDM-equivalent using the conversion factor of 1 g dry tissue weight = 0.81 g AFDM [42]. Thus, we used a value of 1.1 g AFDM in our calculations.

## 3. Results

Both sequestration, via biogenic calcification, and emission, via respiration and calcification, have fallen sharply with the alteration of the CRD estuarine ecosystem. Estimated sequestration from current clam populations is 0.1 mol C m^−2^ yr^−1^, whereas pre-dam abundances imply sequestration of 1.8–4.4 mol C m^−2^ yr^−1^ (table 2, at low and high densities, respectively). Atmospheric emissions due to calcification have also declined by roughly an order of magnitude from 1.3 to 3.1 mol C m^−2^ yr^−1^ (at low and high pre-dam densities, respectively) to 0.07 mol C m^−2^ yr^−1^. Similarly, historic emissions from respiration ranging from 4.9 to 12.2 mol C m^−2^ yr^−1^ (at low and high densities, respectively) have declined to 0.3 mol C m^−2^ yr^−1^. As a consequence, net carbon emissions have decreased from a range of 4.4–10.9 to 0.26 mol C m^−2^ yr^−1^. These values correspond to a cumulative annual reduction in tidal flat carbon emissions ranging from 5.9 × 10^3^ to 15.0 × 10^3 ^t.

**Table 2.** Estimated carbon sequestration and emission for the pre-dam and modern eras. Pre-dam low (50 ind m^−1^) and high (125 ind m^−1^) refer to the number of individuals per square metre inferred from chenier deposits [23]. Emissions via calcification were estimated at salinities of 30 psu and 38.5 psu for the pre-dam and modern eras, respectively. Emissions from respiration were estimated based on an ash-free dry mass of 1.1 g ind^−1^.

				Δcarbon (low, high)	
	pre-dam low (mol C m^−2^ yr^−1^)	pre-dam high (mol C m^−2^ yr^−1^)	modern (mol C m^−2^ yr^−1^)	mol C m^−2^ yr^−1^	t C yr^−1^
sequestration via calcification	1.8	4.4	0.11	1.7, 4.3	2400, 6200
emission via calcification	1.3	3.1	0.068	1.2, 3.0	1700, 4400
emission via respiration	4.9	12.2	0.3	4.6, 11.9	6600, 17 000
net emission	4.4	10.9	0.26	4.1, 10.6	5900, 15 200

## 4. Discussion

The reduction in carbon emissions, by 4.1–10.6 mol C m^−2^ yr^−1^, due to the decline of *M. coloradoensis* populations in the CRD likely corresponds to a large proportional decline in carbon emissions from the estuary as a whole. Borges *et al*. [8] estimated for estuaries from low latitudes (0–30°) and high latitudes (30–60°) that carbon emissions were 17 and 46 mol m^−2^ yr^–1^, respectively. The CRD is located at approximately 31°N, suggesting that the carbon reductions calculated here represent a reduction of roughly 9–23% for the entire estuary. The estimates from Borges *et al*. [8] may, however, overestimate estuarine emissions owing to the abundance (69%; *n* = 16) of high *p*CO_2_ European river systems used to make the estimates [12,53,54]. In contrast, emissions from several ‘high latitude’ estuaries in the United States were reported to be considerably lower: 15–36 mol C m^−2^ yr^−1^ [12,55]. Thus, the reductions reported here might correspond to a decline of up to 70% for annual estuary carbon emissions. This considerable change for the estuary has the potential to significantly alter the CRD ecosystem [16,56] and influence economically important local mariculture [57]. Despite these implications, the conveyance, storage and emission of carbon by rivers—under natural or human-altered conditions—has only recently [6,7,53] factored prominently into assessments of the trade-offs that accompany decisions to store water in reservoirs, to divert it for agricultural and municipal use, or to use it for hydroelectric power generation [58–61]. Even so, these decisions certainly imply different outcomes for the carbon footprint associated with the managed river network [7].

Our calculations were constrained in part by the uncertainties inherent to using palaeoecological data; however, we most likely underestimated the difference in carbon emissions before and after extensive river diversion. The largest uncertainty in our analyses was the estimate of clam density prior to extensive water diversions. Many processes transport (e.g. wave action, currents) and degrade (e.g. fragmentation, dissolution^2^) the remains of organisms after death [25], and the shells preserved in cheniers provide a conservative, lower bound on original density. Additional uncertainty was due to the absence of precise estimates for *M. coloradoensis* AFDM. Despite being congeneric, slight differences in shell morphology between *M. coloradoensis* and *M. edulis* may have introduced minor errors into our calculations. Notwithstanding these considerations, our estimates for pre-dam carbon emission and sequestration are comparable to values reported for other calcifying organisms such as clams [13,15], corals [62], barnacles [63] and brittle stars [64] (table 3). These studies support the validity of our parameter estimates, and strengthen the conclusion that carbon emissions from the molluscan community in the estuary of the CRD have dropped precipitously following the complete appropriation of the river's flow.

**Table 3.** Carbon sequestration and emission for other calcifying systems.

species	sequestration via calcification (mol C m^−2^ yr^−1^)	emission via calcification (mol C m^−2^ yr^−1^)	emission via respiration (mol C m^−2^ yr^−1^)	source
*Potamocorbula amurensis*	2.2	1.5	3.1	Chauvaud *et al*. [13]
*Ruditapes philippinarum*	8.2	5.6	22.7	Mistri & Munari [15]
*Mytilus galloprovincialis*	136.6	86.8	187.8	Munari *et al*. [22]
barnacles	4.8–18.0	3.4–12.7	3.9–14.1	Golléty *et al*. [63]
brittle stars	6.8	4.8	–	Migne *et al*. [64]
corals	15.0	12.0	–	Ware *et al*. [62]

Whereas the reduction in carbon emissions is likely a significant portion of the pre-dam era estuary emissions, the mass is small relative to overall carbon emissions resulting from water management in the southwestern USA. For instance, the United States Bureau of Reclamation uses a 24.3% share of power from the coal-fired Navajo Generating Station to lift Colorado River water to Phoenix and Tucson through the Central Arizona Project, emitting 1.1 × 10^6^ t (approx. 9.2 × 10^10^ mol) of carbon annually (http://ghgdata.epa.gov/ghgp/main.do#). Similarly, Shrestha *et al*. [6] estimated that 1.4 × 10^5^ t (approx. 1.2 × 10^10^ mol) of carbon are emitted annually as a consequence of conveyance of Colorado River water to the Las Vegas valley. By comparison, the reduced carbon emissions at the delta resulting from diverted flow are vastly outweighed by the carbon emissions required to divert that flow.

The estuary emissions reduction may not be significant compared with other carbon emissions related to water management in the Colorado River system; however, extrapolating to a global scale, the mass of reduced carbon emissions becomes much larger. An overview of the world's largest river systems revealed that 172 out of 292 have been diverted by dams and water management [1]. Assuming the conditions in the Colorado River system are representative of the average large river system, then global reductions in carbon emissions associated with molluscan populations are on the magnitude of 1.0 × 10^6^–2.6 × 10^6^ t C yr^−1^, using the low and high estimates reported here. Estimates such as these are often prone to a large degree of uncertainty (±50%) given the tenuous nature of the assumptions behind them [10,65]. Keeping this in mind, the hypothetical reduction in global carbon emissions is at most on the scale of a large power plant (i.e. Navajo Generating Station).

Although modest in comparison with the present-day emissions resulting from river management, the change that we document nonetheless illustrates the need to advance and refine the science to support better accounting of the carbon budgets associated with rivers and water management systems [6,7,66]. Carbon emission from clams is one of many components that contribute to a river's total carbon footprint (figure 1). The complexity of carbon cycling in rivers and estuaries reflects the diverse organisms that inhabit the interwoven components of these systems and understanding these connections will be critical to well-informed planning and policymaking under an uncertain future. For instance, as climate change increases temperatures and the frequency, duration and severity of drought in the southwestern USA [67,68], integrated management of water, energy and ecosystem services is essential. The unintended reduction in net carbon emissions following the decline of mollusc populations in the CRD further demonstrates the need to seek solutions to pressing global challenges that maximize ecosystem services while maintaining ecosystem function as new social priorities emerge or new scientific insight is gained [69–71].

## Supplementary Material

Smith et al. RSOS Supplement One.xlsx

## Footnotes

1 350 µatm was used by Frankignoulle *et al.* [47] to establish the ψ equation and is maintained here. Frankignoulle *et al.* [47] reported an increase in *ψ* as *p*CO_2_ increases, resulting in positive feedback. Consequently, *ψ* is higher today than it was in the past, but 350 µatm remains an appropriate estimate for the entire post-dam construction era (1930–present). In the pre-dam era, *ψ* was likely lower; however, a decrease to 290 µatm (an estimate for the pre-industrial era [47]) results in only a small change (0.04) in *ψ*. Thus, given the small difference and because the pre-dam era also encompassed higher *p*CO_2_ during the industrial era, 350 µatm was also used for pre-dam era calculations.

2 The process of dissolution releases carbon dioxide and contributes to carbon cycling. Because cheniers are subaerial accumulations, however, they are not affected by the same set of destructive processes as assemblages in other marine environments [36]. Thus, shell dissolution via seawater was likely limited. The dissolution that did occur on the tidal flat would have effectively reduced the amount of carbon sequestered in clam shells, resulting in greater net emissions proportionate to total clam abundance today and in the pre-dam era.

## Permission to carry out fieldwork

No fieldwork was conducted for the completion of this study.

## Data accessibility

All data used in this study are reported within. See electronic supplementary material for the raw data on shell mass.
